# Diabetes care in a complex humanitarian emergency setting: a qualitative evaluation

**DOI:** 10.1186/s12913-017-2362-5

**Published:** 2017-06-23

**Authors:** Adrianna Murphy, Michel Biringanine, Bayard Roberts, Beverley Stringer, Pablo Perel, Kiran Jobanputra

**Affiliations:** 1 The Centre for Health and Social Change, Department of Health Services Research and Policy, London School of Hygiene and Tropical Medicine, 15-17 Tavistock Place, WC1H 9SH, London, UK; 2 Médecins Sans Frontières (MSF), Goma, Democratic Republic of Congo; 3 Médecins Sans Frontières (MSF), Manson Unit, 10 Furnival Street, EC4A 1AB, London, UK; 4 Department of Epidemiology and Public Health, London School of Hygiene and Tropical Medicine, Keppel Street, WC1E 7HT, London, UK

**Keywords:** Diabetes mellitus, Treatment, Conflict, Non-communicable diseases NCDs, Humanitarian, Emergency, Low- and middle-income countries LMICs

## Abstract

**Background:**

Evidence is urgently needed from complex emergency settings to support efforts to respond to the increasing burden of diabetes mellitus (DM). We conducted a qualitative study of a new model of DM health care (Integrated Diabetic Clinic within an Outpatient Department [IDC-OPD]) implemented by Médecins Sans Frontières (MSF) in Mweso Hospital in eastern Democratic Republic of Congo (DRC). We aimed to explore patient and provider perspectives on the model in order to identify factors that may support or impede it.

**Methods:**

We used focus group discussions (FGDs; two discussions, each with eight participants) and individual semi-structured qualitative interviews (seven patients and 10 staff) to explore experience of and perspectives on the IDC-OPD. Participants were recruited purposively to represent a range of DM disease severity and staff functions respectively, and to ensure the age and gender distribution was representative of the population of DM patients registered in the clinic. Data were coded in NVivo10© and analysed using an inductive thematic approach.

**Results:**

There appears to be little awareness surrounding DM in patient communities, resulting in delays presenting to hospital. Patients describe their first reactions to symptoms as fear and confusion, often assuming symptoms are of another disease (e.g. HIV/AIDS). They often express disbelief that they could have DM (e.g. stating DM is a ‘rich man’s disease’) and lack acceptance that there is no cure. Patients experienced difficulty travelling to appointments, exacerbated by flare-ups in the conflict. Providing psycho-social and sensitisation activities in a group setting appears to offer an opportunity for patients to support each other in their effort to adhere to drug treatment and follow-up appointments. All patients reported great difficulty in adhering to the recommended diet, which was viewed as unaffordable and unavailable, and fear that this would be the biggest obstacle to maintaining their drug treatment (as treatment must be taken with food).

**Conclusion:**

Our findings emphasize the importance of community awareness of DM and the value of treatment support, including psychosocial and educational support to DM patients and their families, and culturally sensitive, low-cost dietary advice, to ensuring the adoption and maintenance of DM treatment.

**Electronic supplementary material:**

The online version of this article (doi:10.1186/s12913-017-2362-5) contains supplementary material, which is available to authorized users.

## Background

The global burden of non-communicable diseases (NCDs) is growing rapidly worldwide, and particularly in low-and middle-income countries (LMICs) [1, 2]. This is especially true of diabetes mellitus (DM), one of the most common NCDs, which has increased globally by almost 45% since 1990 [3]. The largest proportional increase expected in adult diabetes cases, 90.5% by 2030, is expected in the World Health Organization (WHO) African Region [4].

The risk of mortality and morbidity from DM is significant, both from acute events and from long-term micro-and macro-vascular complications. Early detection and consistent treatment with drug therapy can significantly reduce these risks [5, 6]. Yet, in countries at all levels of development, a substantial proportion of people with DM go undetected and untreated [7]. In countries with poorly co-ordinated and fragmented health systems, levels of premature mortality from DM are particularly high [8–10]. These problems are compounded in complex humanitarian emergency settings where health systems have broken down [9] and the accessibility of primary care, availability of trained staff, and co-ordination between health facilities required for effective DM interventions are compromised.

Despite the growing issue of DM and other NCDs globally, and their increased relevance among conflict-affected populations [11], there is a dearth of evidence on how interventions should be delivered in complex emergency settings [12], and little attention has been given to NCDs by humanitarian actors [13, 14]. While the experience of delivering DM care in a conflict setting in Mali has been described [14], and the effectiveness of a DM programme in Palestine assessed [15–17], the factors impeding or supporting the effectiveness of such programmes have not been evaluated. Such evidence is urgently needed from complex emergency settings to support the efforts of humanitarian agencies in responding to DM. We conducted a qualitative study of a new model of DM health care implemented by Médecins Sans Frontières (MSF) in the Democratic Republic of Congo (DRC). We aimed to explore the patient and provider perspectives on the model in order to identify factors that may support or impede it.

## Methods

### Research setting

Since 2008, MSF has offered primary and secondary health care in Mweso health zone in the Health District of Masisi, North Kivu Province, eastern DRC. Mweso is a rural, low-income region affected by the conflict between government and rebel forces that has persisted since the end of the Second Congo War in 2003. Mweso zone has an estimated population of 365,000 people, including 17 camps for internally displaced people. MSF, in collaboration with the local Ministry of Health (MoH), supports health services in the hospital in Mweso and in four of 23 primary health care clinics in Masisi.

Mweso Hospital serves a catchment area of an estimated 145,000 people, but the realistic number of beneficiaries in the immediate catchment area is likely closer to 65,000. There are 130,000 out-patient consultations per year and 5000 in-patient admissions. There are no data on the prevalence of diabetes in this specific population, but the International Diabetes Federation estimates a DM prevalence of 5.3% for DRC overall [18].

Until March 2015, Mweso Hospital routinely treated DM patients presenting with acute complications. However, there was a lack of clinical guidance, standard operating procedures (SOPs), and appropriately trained staff. In March 2015, a new model of DM care was implemented by MSF. The Integrated Diabetic Clinic within an Outpatient Department (IDC-OPD) is based on simplified context-adapted clinical guidelines, clinical SOPs, adapted patient counselling and support materials, and one-off staff training by a diabetologist. IDC-OPD is a nurse-led, multi-disciplinary model of care adapted to the specific setting of Mweso Hospital. The clinical guidelines were developed for the Mweso setting based on WHO, International Diabetes Association, National Institute of Clinical Excellence and MSF guidelines, with input from a diabetes specialist and experienced MSF clinicians. Diabetes educational tools were developed for Mweso based on tools developed by Diabetsante (Mali).

The service is managed by a nurse supervisor who oversees the running of the clinic. Care on clinic days is also provided by a nursing assistant, two doctors who provide medical support and see referred patients, a nutritionist, an information, education and counselling officer, and a psycho-social support officer. Most patients enter the clinic cohort after discharge from the in-patient ward, where DM is usually diagnosed following acute presentation. A small proportion are diagnosed in Mweso out-patient-department or referred from external clinics. Every patient is recorded in the clinic register and has an individual patient file. Outpatient clinical procedures involve new patient assessment, monthly nurse-led follow-up appointments, and 6-month medical review. More details on the model are in Additional file 1: Appendix A.

At the time this study was conducted there were 107 active patients of 177 ever registered. The median age of the cohort was 45 years, and 19 were younger than 20 years. Based on the large proportion of younger patients with high insulin requirements, who present with recurrent non-ketotic hyperglycaemia (and early onset of chronic complications such as blindness), we suspect that a significant proportion of the cohort have malnutrition-modulated diabetes. However, the majority of the cohort have typical type 2 disease, with a handful of type 1 cases.

### Study design

We conducted a qualitative study as part of a mixed methods evaluation using the RE-AIM framework [19]. The RE-AIM framework acknowledges that public health interventions act on multiple levels (e.g. individual, health-care provider, institutional) and that their impact relies on the combined effect on five evaluative dimensions: Reach (proportion of population affected by the programme); Efficacy (positive and negative outcomes of the programme); Adoption (degree of participation in programme); Implementation (degree to which programme is implemented as intended); and Maintenance (institutionalization of the programme). The qualitative element of RE-AIM seeks to identify factors that may support or impede the achievement of these dimensions with respect to the IDC-OPD programme. In addition to the qualitative study, the full evaluation of the IDC-OPD includes quantitative analysis of routine programmatic and costing data, and a cross-sectional survey of prevalence and service use. The findings of those studies will be reported separately.

### Data collection

We used a combination of focus group discussions (FGDs) and individual semi-structured qualitative interviews to explore patient and staff experience of and perspectives on the IDC-OPD.

We held two FGDs with eight patients each, to assess the appropriateness of the general categories included in our topic guide. We then conducted semi-structured individual interviews, to enable in-depth exploration of an individual’s perception of a particular phenomenon [20]. Patients were recruited purposively to represent a range of DM disease severity and staff functions respectively, and to ensure the age and gender distribution of the sample was broadly representative of the population of DM patients registered in the clinic. All staff involved in the DM clinic were recruited for interviews. (Staff involved in the clinic happened to be all male, which, based on our experience, is not uncommon in DRC). Eligible participants were provided with a full explanation of the study objectives and given an information sheet with these details. It was also explained to them that their participation in the study was strictly voluntary and would not impact on their access to care. None of the invited eligible participants refused to participate in the study and all participants signed an informed consent form. We conducted individual interviews until data saturation was achieved (i.e. additional interviews did not uncover new themes related to our research question). The final number of individual interview participants was seven patients and 10 staff members. (Table 1) As we were interested in over-arching themes, or meta-themes, we were able to achieve saturation with a smaller sample size than would normally be needed for finer-grained themes [21].

**Table 1:** Study participants

Patients	Approximate age	Gender
P01	16	male
P02	70	female
P03	46	male
P04	54	female
P05	58	male
P06	60	male
P07	49	male
Staff	Role	Gender
S01	Consultation nurse	male
S02	Psycho-social support officer	male
S03	Information, education and counselling officer	male
S04	Local MoH doctor	male
S05	Nurse supervisor	male
S06	Mental health officer	male
S07	Nutritionist	male
S08	MSF doctor	male
S09	MSF doctor	male
S10	MSF doctor	male

*MoH* Ministry of Health, *MSF* Médecins Sans Frontières

The topic guides first covered background information with regard to the patients’ experience of DM, or the staff member’s role in the IDC-OPD, and then included prompts related to each of the five RE-AIM dimensions (e.g. barriers to accessing testing for DM, information and support provided to patients to encourage adherence, challenges to maintaining adherence, unintended negative consequences of treatment). The complete final English version of the topic guides for the FGDs, patient interviews, and staff interviews can be found in Additional file 1: Appendix B, C, and D respectively. All interviews were conducted in a private room and in the participant’s spoken language (Swahili, French, or English), by a trained researcher. Interviews in Swahili and French were conducted by MB (male, Assistant to the Humanitarian Affairs Office at MSF Goma); interviews in English were conducted by AM (female, public health researcher at the London School of Hygiene and Tropical Medicine). Interviews were audio-recorded, transcribed, and, where necessary, translated into English by trained third-party researchers not otherwise involved in the study.

### Analysis

Data were coded in NVivo10© and analysed by AM using an inductive thematic approach. Inductive thematic analysis involves coding data for emerging themes and concepts without trying to accommodate a hypothetical framework, and is thus ‘data driven’ and reflexive [22, 23]. Analysis followed the steps to thematic analysis outlined by Braun and Clarke [22]. First, transcripts were read in their entirety and initial open codes (e.g. ‘Fear of diagnosis‘, ‘Staff pride in programme‘) were generated. Second, all coded excerpts were organised into a process of selective coding where core codes were developed applying constant comparative analyses toward categories (*e.g.* ‘Acceptance of DM treatment', ‘Staff investment and capacity”). Finally, organic identification and development of latent patterns, to generate themes applying principles of grounded theory (meaning emergent codes and themes were based in the data derived from interviews) were checked with reflexive practice to ensure against the insertion of preconceived assumptions. These themes were related back to the research question and to existing literature. Negative cases or exceptions were examined to test emerging themes and to analyse why these cases were different. Findings were scrutinized by two team members to enhance inter-rater reliability and analytic credibility. We report our findings following the ‘Consolidated Criteria for Reporting Qualitative Research’ checklist for transparency [24].

## Results

The final sample of study participants for patients (P) and staff (S) are presented in Table 1. Two dominant themes emerged, highlighting factors that may support or impede the reach, efficacy, adoption, implementation, and maintenance of the IDC-OPD model: i) community awareness, knowledge and family support; and ii) sustainable treatment approach. These themes and the categories and codes that they were drawn from are shown in Table 2. We elaborate on key findings within each of these themes below.

**Table 2:** Coding framework

Theme	Category	Codes	
		Undermine programme effectiveness	Promote programme effectiveness
Community awareness, knowledge and family support	Acceptance of DM Adherence to DM treatment Knowledge Accessibility of treatment	Ignorance regarding DM Word-of-mouth only DM awareness Reliance on traditional healers Fear of diagnosis Feeling of emasculation among men relating to diagnosis Recommended foods difficult to obtain, expensive Transportation to clinic challenging in conflict Long distance to clinic Perception of traditional medicines as more effective	Acceptance of chronic nature of disease View of medicine as the difference between life and death Awareness of consequences of not treating Support of family in DM diet and treatment adherence Bonding with other DM patients Appreciation of importance of regular appointments Patient education as empowerment
Sustainable treatment approach	Implementation of DM programme Staff investment and capacity Ownership and certainty of programme Maintenance of DM programme	Heavy workload for staff compromises quality Low expectations for programme capacity No dedicated programme resources Dependence on NGO to maintain programme Low institutional investment in programme	Staff pride in programme Motivation from increased skills and responsibility

*DM* diabetes mellitus, *NGO* non-governmental organisation

### Community awareness, knowledge, and family support: *empowerment, acceptance, and adherence to DM treatment*

There appears to be very little awareness surrounding DM in patient communities, resulting in delays before patients present to the hospital with symptoms. Patients describe their first reactions to their symptoms as fear and confusion, often assuming these are symptoms of another disease of which they are more aware (e.g. HIV/AIDS).

*" I started feeling thirsty for water, then I started losing weight, people started saying that I have AIDS. So I started feeling scared, people were saying that I would die soon.....I didn’t know what was affecting me and I would have just died. They saw the symptoms because I lost a lot of weight and many people were confused. If nobody would have told me I would have continued the same way until death." (P01, male)*

Seeking care from a traditional healer in the first instance appears common, and those who eventually make it to the IDC-OPD often only do so because they hear about the programme by chance, suggesting that there is likely a much larger proportion of diabetes sufferers who are not being reached by the IDC-OPD.

*"I was home then I started feeling bad. I was passing urine frequently and felt weak.*

*I went to seek help from herbalists and was given herbal medicine. I kept taking it but was not improving. Later I met a friend who advised me to come to Mweso." (P02, female)*

The reaction described by patients upon receiving a DM diagnosis suggests fear and denial of their condition.

*"I thought I was going to die. I despaired and felt hopeless. They said it’s incurable. I just knew I would die." (P04, female)*

*"There and then I felt fear. I had been told diabetes is for rich people. But now me, a poor man? I was afraid. I was afraid because diabetes is an expensive illness for the rich. What was I supposed to do in my poverty?" (P06, male)*

They often express disbelief that they could have DM (e.g. that DM is a ‘rich man’s disease’) and lack acceptance that there is no cure for their condition. The patients that we interviewed represent those that have overcome this fear and disbelief to return to the IDC-OPD for treatment; however, interviews with the staff responsible for the IDC-OPD clinic suggest that a significant number of patients do not return to the clinic after being diagnosed, often seeking a ‘cure’ from traditional healers rather than facing the chronic nature of their disease.

*"After diagnosis...they (the patients) do not want to accept that 'ok, this is the problem I have'. There is also a feeling of guilt, they will feel guilty 'where did I pick up this disease?'...Commonly, when we diagnosis a person, they exhibit these feelings. Either they will refuse, 'no this is not possible, how did I get this disease? Where did I get it? Did she give me this disease?' Because some patients say 'maybe it is someone who contaminated me with this disease.' Because, from the beginning, they do not have good information about the disease." (S06)*

*"When they go back home, the patient may well be influenced by the traditional practitioners, by telling him that he will cure him. And then, he thinks he must be cured. Why wouldn’t he? He believes the healer. We say something different; we say it is an incurable disease yet it is a disease that you can control. It’s easier for him to listen to the other message that gives him hope for cure. This is his ultimate goal: cure. " (S02)*

*"Unfortunately, there was a case who went for indigenous products... This is just one example we give to the other patients..He thought he was cured with indigenous products; we had screened him and started him on pharmaceutical medication and then he was sold some tradi-practitioners treat. He left and remained stable for around two years, I don’t know how, miraculously… But complications set in and when he came in, he had such bruises we had to amputate him. Now he is in the clinic. "(S08)*

Providing psycho-social and sensitisation activities in a group setting appears to offer an opportunity for patients to support each other in their effort to adhere to drug treatment and follow-up appointments. Patients reported creating relationships with others in the IDC-OPD and coming to clinic appointments together.

*"At the beginning, it may frustrate the patient. But, with continued visits and because he meets other patients, at the second, third visit, he feels much better. He is not alone. The other patients are doing fine. This helps him continuing with what we provide in the programme." (S04)*

Patients who had come to the clinic also reported feeling that their family had accepted their condition and were helping them to manage it, which suggests that family support is an important element of adhering to dietary treatment regimens. Yet interviews with both patients and staff suggested that ensuring family support for, and understanding of, DM is challenging in early stages post-diagnosis and may act as a roadblock to maintenance of the IDC-OPD if not achieved. At times the threat to family support appears to be related to gender roles in the home, with male patients expressing anxiety on the part of their family about losing the family breadwinner. According to interviews with staff, the temptation among patients to seek a more effective ‘cure’ for diabetes outside the IDC-OPD persists even after treatment at the clinic is initiated, resulting in patient attrition. The themes of patient empowerment from group counselling activities and family support are illustrated in quotations from staff and patient interviews below.

*Interviewer(I): "How about your wife, how do you think she is handling your condition?"*

*Patient (P): "She gets discouraged. She wonders for how long this will continue. She figures I am already gone, living on medicines all my life. It brings her worry. But I cannot stop injecting myself....The diet, it brings a lot of strife. Because she has to prepare her food with the children, then prepare mine. It brings many quarrels. We just live by the grace of God." (P06, male)*

All patients interviewed in our research reported great difficulty in adhering to the diet that is recommended to them by the IDC-OPD nutrition team. Interviews revealed feelings of helplessness in trying to maintain a diet that is viewed as unaffordable and unavailable, and fear that this will be the biggest obstacle to maintaining their drug treatment (as treatment must be taken with food).

*"I would follow the diet happily, but unfortunately the foods I am asked to eat are very difficult to find. We have the red bananas which I can only eat a little of. The right food to eat is difficult to get. It’s not easily available where I come from." (PO3, male)*

*P: "I do my best to get food so as to take my medicine. But, diabetes is a rich man’s disease"*

*I: "Do you stop taking medication when you have no food? "*

*P: "Yes, I sometimes stop. This disease is expensive. "(P04, female)*

This challenge is compounded by the fact that patients feel their dietary restrictions cause a burden to their households, who have to create separate meals for them.

*" It (this disease) is difficult because it requires two meals in the family. Sometimes you have no money to afford the meals. It’s more expensive and the income is less. Because they have to separate my meals. People who inject, they need to eat; they need to have certainty of meals. When they get home they worry, thinking they may die due to lack of food. Majority of us are very poor, sometime you find you have nothing, you get discouraged because you have medicines but not the food, especially the ones who inject. You must eat before injecting" (P05, male)*

The Mweso IDC-OPD is the only DM clinic in North Kivu, DRC. As such, many patients travel to Mweso from far distances, sometimes up to 4 or 5 h away to acquire medications. Travel distance to the clinic appears to present an obstacle to patients maintaining clinic appointments and acquiring medication for treatment adherence,

*"I: How do you find coming here for every appointment to collect medicines? Is it easy for you? Do you encounter any difficulties?*

*P: It’s difficult, every day selling bitter vegetables and paying the fare to this place regularly. It’s not easy. It’s tiring. You need flour and vegetables for food, yet you are forced to have money for your fare here." (P02, female)*

*"I come for appointments from (place name). When I have money, I pay for a ride. Like today, I walked to Kichanga then found someone assisted me with some money and I took transport to here. When I have money I pay fare and come, otherwise I walk...Walking from (place name) to Mweso takes four hours. It takes will power to come because of the expenses. It’s a long distance. It takes sacrifice because I never want to miss my appointment. " (P06, male)*

Travel to clinic appointments appears to be particularly challenging at times of conflict in the region. North Kivu is a region of instability, with regular incidences of violent conflict. During periods of fighting, travel to Mweso from far distances becomes even more difficult than usual, if not impossible.

*"Some of us come from very far where you need transport here, for example (place name). Is it possible for you (MSF) to arrange for us to collect medicine close to where we come from? Because sometime the roads are blocked by fighting and medicine is finished and you have an appointment to come to Mweso and you have no way of coming. Is there a way you can help us? Because there is a dispensary at (place name)." (P03, male)*

*“If they could bring the medicines to (place name of home village) it would make it easier (collecting medicines). Because sometimes the roads are bad and people can get attacked. Like when some people were attached that time in Au Baibe.” (P02, female)*

### Sustainable treatment approach

A second theme - *Sustainable treatment approach* - emerged predominantly from interviews with staff. The staff interviewed for this study were eager to discuss their role in the programme and in following protocols, and were animated in describing its successes. Pride in their achievements with the programme appear integral in creating and maintaining their enthusiasm for continuing their efforts in the IDC-OPD, despite major challenges with time and human resources.

*" Regarding the support, it has improved a lot. Because before, when we did not have this clinic, there were diabetic patients who would arrive either in hypo/hyperglycaemia, we took them up only within the boundaries of the little bits of medication we had available within the ward. There were some of these drugs that were not available at the big pharmacy. But since we started this support, we have seen, I saw, that there is a follow-up and by the way we are very happy....I am thankful also because we follow-up diabetic pregnant women! Really, with the support, I can see that it is really, 100%, something good. " (S01)*

*" The quality of care is the best here. When each patient arrives to the hospital, first of all, he is well welcomed. We try to test his glucose; we check his weight, his blood pressure. We try to do a nurse consultation, ask the patient if he has any particular complaint during his stay at home. Then....once we see that there a positive aspect that allows us to judge that the patient can see the physician for a medical consultation, we guide him and transfer him to a medical consultation for an appropriate management. And if we see that the patient has a stable glucose rate, no particular problem, we the nurses, we try to conduct the treatment of the patient and then we provide him with the next appointment of the Diabetic clinic. (S05)*

At the time of our research, staff had only received one formal capacity-building session and it had been several months since this had occurred. Local staff remained dependent on MSF staff and repeatedly emphasized a desire for further capacity building, suggesting that failure to deliver such support may result in reduction of motivation and commitment to the IDC-OPD.

*S: "With regards to diet, if the partner (MSF) could help us with a nutrition programme and nutritionists available to follow up the patients with us and help them become more disciplined with their diet, it would be helpful...Of course, we have the economic problem because the diet we propose is pricy for the patients. It is an additional obstacle. "*

*I:" Thank you very much. Do you have anything to add or suggest anything to improve the programme?"*

*S:" OK. It is about our development and continued education. The activity is quite new and we do need an on-going training....We need regular updates. If you (MSF)are conducting such programmes in other provinces or countries, it would be helpful for us to see them....The problem we have is that we are not always confident about what we do. We feel sometimes that we have a limited knowledge or experience....We have no clue about our weaknesses when it comes to stabilising the patient. We still have no solution to that." (S04)*

*"Thankfully we have the doctors who are here and helping us try to change...you know, in scientific life, man cannot stay immobile. Man needs to change. Needs to get information. And these pieces of information will help improve the quality of care both from a staff and a patient's point of view...when man limit ourselves to only routine, routine, routine, the consequence will not only be suffered by him, but also by the patient. We are trying to alter the system! " (S01)*

The IDC-OPD programme in Mweso was initiated by MSF, to respond to the need expressed by both MSF and MoH staff at Mweso hospital, for support in responding to increased numbers of DM patients. DRC’s national policy on DM is only partially implemented and the country’s framework for monitoring and surveillance of DM is not routinely implemented and receives little political support [25]. The MoH looks to MSF for guidance on programme protocols and there appears to be no initiative on the part of MoH to take responsibility for further development of protocols or capacity-building programmes. This dependence on MSF appears to create insecurity among staff about the future of the programme, given the uncertainty about the duration of MSF’s presence in the region.

*"For example, we need to know specifically that the programme will last one or two years, or more… If we have this guarantee, we have visibility and we can come up with a plan to control and manage the patients. We would know what to share with the patients. Sometimes we have reserves regarding the education, because we project things in the future. We say, for example: “next year or the next six months you will be this and that and you will have to do this and that… “ While even us, we don’t have the guarantee that the programme will be there to help the patient and do a proper follow-up. "(S02)*

## Discussion

A review of the literature suggests that this is the first study to identify factors that impede or support the effectiveness of the DM programme in a complex humanitarian setting in Sub-Saharan Africa. Two over-arching themes – *community awareness, knowledge, and family support*; and *sustainable treatment approach* – emerged and highlighted factors that may promote or impede the reach, efficacy, adoption, implementation, and maintenance of the IDC-OPD model.

The first of these themes points to the importance of community awareness to the effectiveness of DM interventions. These findings are consistent with existing evidence from LMICs highlighting the importance of community awareness for improving not only the reach of evidence-based interventions such as HIV [26], breast cancer [27], and cervical cancer [28] screening, but also the efficacy, adoption, and maintenance of HIV interventions through community mobilisation, or the involvement of the community in the planning, implementation, and ownership of the intervention to ensure its relevance and accessibility [29].

Our findings also emphasize the value of treatment support, including psychosocial and educational support to DM patients and their families, and culturally sensitive, low-cost dietary advice, to ensuring the adoption and maintenance of DM treatment. Patient and family psycho-social support and education have proven to be integral to ensuring acceptance of long-term or chronic illnesses and persistent adherence to medication. The recent experience of MSF in Kenya with Medical Adherence Clubs for NCD and HIV patients (groups of patients who meet every 3 months to collect pre-packaged prescription medicines, engage in health education and discussion and have their weight and blood pressure measured) suggests that such models of care are acceptable to patients and staff, who perceive them as time-saving [30]. Likely the largest body of evidence of the effectiveness of psychosocial education and support comes from research on HIV and TB, where community patient groups [31], social support [32], and patient and family education activities [30, 33] have been found to improve treatment adoption and maintenance. New interventions that adapt these lessons to NCD programmes in LMIC are being trialled. For example, the IMPACT-AF trial [34] is testing the effectiveness of a customized intervention for increasing adherence to therapy among patients with atrial fibrillation (a major risk factor for stroke) in five LMICs. In India the intervention will involve training non-physician health workers to educate patients in AF and symptoms of stroke, and the importance of adhering to medication to prevent stroke, while identifying non-adherent patients and addressing the barriers to adherence that they experience. Diaries are given to patients to allow them to record days when they take medications, and included educational content. While these interventions are currently being implemented in non-crisis-affected settings, the results of their evaluations will nonetheless be relevant to most contexts where patient knowledge is low and access to health facilities and highly-trained physicians is difficult.

Similarly, dietary restrictions have been identified as a potential barrier to adhering to antiretroviral therapy, [35] in particular in the context of food insecurity [36]. Specific to DM, research from low-income populations in higher-income countries such as the USA suggest that limited food environments, limited and abstract communication from clinicians, short term losses vs. gains and stress from poverty all contributed to low adherence to DM dietary regimes in these contexts, and highlight the need for customized dietary advice [37].

While our findings are consistent with evidence from non-crisis-affected low-income settings, they also contribute to this body of knowledge by identifying specific factors that may be particularly important to consider in complex humanitarian emergencies. Our findings regarding the challenges faced by patients in accessing treatment regularly highlight two elements that may ensure successful adoption, implementation, and maintenance of a DM or other chronic NCD treatment programme - decentralization of treatment provision and reduced appointment frequency. These factors are relevant to any setting where health facilities are distant and difficult to reach, but as shown in our interviews, in conflict-affected settings, when faced with the possibility of travelling through conflict zones, patients opt to forego treatment. Lessons from research on adherence to antiretroviral treatment for HIV provides useful lessons, suggesting that community-based care models are effective in reducing patient attrition [38, 39].

The second overarching theme that emerged from our interviews – *Sustainable treatment approach* – is relevant to many development contexts, where local ownership and capacity-building have been identified as vital to ensuring health systems strengthening and sustainability of health programmes [40]. With DM specifically, these factors may have a greater impact on treatment access than do issues of pure affordability and availability of medicine [41]. Our findings support the argument that ensuring a sustainable treatment approach through local programme ownership and capacity building is also important to consider in complex humanitarian emergencies, where interventions are initially implemented and then heavily supported by humanitarian agencies such as MSF. Agencies acting in humanitarian crises traditionally keep ‘beneficaries’ as their unique target group, rather than health systems, As such, there are times when they do not work within the health system at all (e.g. such as when, for example, MSF responds to a cholera outbreak in South Sudan where the health system is essentially absent). However, our findings suggest that where impact for beneficiaries would be increased through engagement with the health system, such as in chronic disease programmes (HIV, NCDs) in protracted crises, meaningful engagement with the health system is necessary to ensure sustainability. These findings reflect current discussion around the role of humanitarian agencies in health systems strengthening, and specifically how to balance achievement of quick results with longer-term maintenance of health programmes [42–44]. While strong arguments exist for the value of longer-term thinking in the case of NCD interventions in complex humanitarian emergencies [45], thus far little guidance exists as to how to realize these concepts in such settings. Some lessons can be drawn from successful experiences of MSF and others in the treatment of HIV [46]. Effective strategies may include: ensuring staff (including MoH staff) have a solid grounding in the basics of disease management through regular trainings; establishing partnerships with local government and other health authorities, governed by memoranda of understanding, to integrate the programme with other health services, including with drug supply chains; ensuring buffer stocks of drugs rather than outright provision; and scale-up and incorporation of successful programme elements into national guidelines, as has been recommended with community ART distribution programmes for HIV [47, 48].

### Limitations

Our findings should be interpreted in light of some limitations. First, we were only able to recruit patients that were present at the clinic in the period that the study took place. As such, the findings may not be generalizable to those with DM who did not come to the clinic in that period. These individuals may have shared important information on barriers to adherence to treatment. However, these themes are also likely to have been captured in the data from included patients, as some of these had at some point not regularly kept appointments or adhered to treatment. Data from interviews with staff, who interacted with and observed the behaviour of all registered DM patients, would also have shed light on barriers experienced by non-adherent patients. Second, it is possible that interviewed patients or staff may have responded to our interview questions with what they believed to be the response that the interviewer wanted to hear. However, we feel that this is unlikely, as it was made clear to all participants that neither of the interviewers (AM and MB) were involved in design or provision of the DM programme and all partipants appeared to respond openly and naturally during the interviews. Finally, while the themes that we found may be generalizable to the larger population served by this programme or other similar contexts, they may not generalize to DM programmes in dissimilar settings.

## Conclusion

Our study suggests that the effectiveness of treatment models for DM in complex humanitarian emergencies can be improved by addressing community awareness, patient, and family knowledge and psychosocial support, appropriateness of dietary guidelines, decentralization of treatment and ownership and capacity-building of local staff. Our findings highlight the potential role agencies acting in humanitarian crises can play in supporting local health systems to ensure sustainability of health care delivery for chronic conditions. While the factors that we identified in our study are similar to those that are relevant in any low-income setting, they are of particular importance where access to health facilities is impeded and implementing agencies are only present in the short-term. Although our findings are specific to the MSF-implemented IDC-OPD, they provide important insights that are likely to be applicable to NCD interventions in other similar contexts.

## Additional file

Additional file 1: Appendix A: Mweso DM Programme Clinical Guidelines and Standard Operating Procedures. Appendix B: Topic guide for focus group discussions with diabetes patients. Appendix C: Topic guide for semi-structured interviews with diabetes patients. Appendix D: Topic guide for semi-structured interviews with diabetes health care providers. (DOCX 138 kb)

## Abbreviations

DM: Diabetes mellitus
DRC: Democratic Republic of Congo
IDC-OPD: Integrated Diabetic Clinic within an Outpatient Department
LMIC: Low- and middle-income country
MSF-OCA: Médecins Sans Frontières Operational Centre Amsterdam
NCD: Non-communicable disease
RE-AIM: Reach, Efficacy, Adoption, Implementation, Maintenance
WHO: World Health Organization.
