# Supplementary material for: Diabetes care in a complex humanitarian emergency setting: a qualitative evaluation
Source: BMC Health Serv Res. 2017 Jun 23;17:431. doi: 10.1186/s12913-017-2362-5 (PMC5481869; doi:10.1186/s12913-017-2362-5)
Supplement: Additional file 1: — Appendix A: Mweso DM Programme Clinical Guidelines and Standard Operating Procedures. Appendix B: Topic guide for focus group discussions with diabetes patients. Appendix C: Topic guide for semi-structured interviews with diabetes patients. Appendix D: Topic guide for semi-structured interviews with diabetes health care providers. (DOCX 138 kb) [file 12913_2017_2362_MOESM1_ESM.docx]

**Appendix A: Mweso DM Programme Clinical Guidelines and SOPs**

Specific clinical guidelines were developed for the Mweso setting, based on World Health Organization (WHO), International Diabetes Association, National Institute of Clinical Excellence and MSF guidelines, with input from a diabetes specialist and experienced clinicians within MSF. These guidelines cover diagnosis of diabetes, out-patient care for diabetes, insulin initiation and adjustment, management of diabetic emergencies, management of surgical and other hospitalised patients with stable diabetes, and management of gestational diabetes. These guidelines were converted into clinical standard operating procedures which are displaced in hospital departments. An important element of these guidelines was the emphasis on distinguishing type 1 from type 2 diabetes, and prioritising treatment with oral hypoglycaemic (OHG) therapy for type 2 diabetes, moving on to insulin therapy only if acceptable glycaemic control (FPG<200mg/dl) is not achieved on OHGs (typically after 6 months).

Diagnosis of diabetes mellitus is based on WHO Guidelines. Either:

- Random plasma glucose (RPG) > 11.1mmol/l (200 mg/dl) on a minimum of two occasions. Random is defined as the glucose concentration any time of the day without regard to the time since the last meal. OR
- Fasting plasma glucose (FPG) > 7.0 mmol/L (126 mg/dl) on two occasions. Fasting is defined as no caloric intake for at least the past 8h. If the results are discordant, the test should be repeated, and the diagnosis will be made on basis of this value

Diabetes educational tools were developed for Mweso based on tools developed by Diabetsante (Mali), adapted to the context of North Kivu . These tools are mostly visual and cover diabetes self-management, acute and chronic complications, diet, treatment (insulin and tablets), pregnancy, daily foot care, how to recognise symptoms of hyperglycemias and hypoglycaemia, store insulin, and safe injection technique. Since literacy levels are very low in Mweso zone, the tools are designed for use by medical and information, education and counselling (IEC) staff rather than for patient self-education. Therapeutic education is provided by the diabetes nurse and the IEC team on a group and 1:1 basis on every clinic day.

The diabetes nurse also coordinates the activities of the psycho-social team who can provide 1:1 support for patients referred by the nurse, as well as support groups at the weekly clinics. At these groups, patients are asked to describe what is concerning them; the facilitator may ask about available family support and resources that will help the participants and the facilitator to design a realistic self-management plan. Patients with severe coping problems, signs of mental illness, substance misuse, or cognitive decline are followed up on a 1:1 basis.

A nutritionist attends the clinic each week, also providing group education and focused 1:1 education for patients referred by the nurse. The nutritionist advises a balanced diet based on locally available foods and matched to the individual needs of patients; visual support tools have been developed specifically for the setting. The nutritionist also sees any patient with a body mass index (BMI)<16 km/m^2^, for provision of food support according to standard hospital procedures.

**Appendix B: Topic guide for focus group discussions with diabetes patients**

| **Key area** | **Themes** | **Question** |
| --- | --- | --- |
| **Introduction** | Study aim and agencies involved  Why invited to participate  Consent & any questions |  |
| **Participant background** | Getting to know each other + building rapport | Could you tell us a bit about yourself? *Prompt: e.g profession, what area live in, when you were first diagnosed with diabetes?* |
| **Reach** | Access  Barriers to testing and diagnosis  Ways of reducing barriers | What do you know about diabetes? *Prompt – e.g. causes, types, who gets it, treatment*  Could you tell me about how you came to learn about diabetes? *Prompt – e.g. from friends/family, from the radio (or other media), when diagnosed at hospital.*  What were you told about diabetes when you were diagnosed through the hospital diabetes service? *Prompt – probe understanding of diabetes such as causes, risks and its management (medication and diet).*  What do you think might prevent people testing for diabetes? *Prompt:lack of knowledge, lack of services, costs, time, quality of services, stigma etc.*  How could knowledge of diabetes be improved among the populations? *Prompt – outreach, radio, health workers etc*  How could access to testing for diabetes be improved? |
| **Adoption and implementation** | Information  Support | How did you feel when you got the diabetes test result? *Prompt: counselling/support experience. Prompt: subsequent days/weeks experience*  Who did you talk to about the result? *Prompt: E.g. family members, friends.*  What were you told about diabetes care after your result (by the diabetes staff)? *Prompt: medicine types and usage, managing medicines, diet changes, risks and symptoms, frequency of check-ups etc.*  What sources of support did you receive? *Prompt: emotional support from family/friends, information support from health workers, psychosocial support from health workers.*  What made it easy or difficult for you to access care?  How acceptable do you find the diabetes treatment. *Prompt: e.g. logistically, socially, culturally etc.* |
| **Maintenance** | Challenges  Supportive factors  To support adoption and implementation | What have been the main challenges in maintaining your medical treatment for diabetes? *Prompt: time, costs, information, drug supply, stigma/shame etc.*  What have been the main challenges for altering your diet? *Prompt: information, costs, support*  What could have made accessing care easier for you? *Prompt: e.g. information given – content and way it was delivered; costs; type and quality of care and support; focus on role of the diabetes programme/services.* |
| **Efficacy** | Unintended consequences  Benefits | What have been the negative consequences of taking diabetes treatment? *Prompt: physical, psychological, costs, time.*  What have been the benefits of receiving diabetes treatment? *Prompt: e.g. physical, psychological, social, economic.* |
| **Thanks and**  **close** | Anything else to add  Questions  Thanks, feedback info | Anything else to add on topic that we haven’t discussed today?  Any questions for me?  Feedback again on how the discussion will be used and fed back. |

**Appendix C: Topic guide for semi-structured interviews with diabetes patients**

| **Key area** | **Themes** | **Example Questions** |
| --- | --- | --- |
| **Introduction** | - Review study aim - Why invited to participate - Consent |  |
| **Participant Background** | - Diabetes knowledge - Experience with diabetes, background - Expectations | - What disease do you have? - Tell me about your experience living with diabetes, your story. - What do people in your community think about diabetes? - How did you get diagnosed? Have you been coming to the clinic since then? - Before you started treatment, how did you feel? What about after starting treatment? - Do you think that one day you will get better? |
| **Reach** | - Knowledge in community - Access to testing for diabetes - Barriers to testing | - Do you think a lot of people have diabetes in your community? - What do you think prevents people from being tested for diabetes? - What do you think would make it easier for people to get tested for diabetes? |
| **Adoption and implementation** | - Information and other support provided - Adjusting to condition | - How did you feel when you received your diagnosis? - Was any information provided to you about your condition? What other support would you have liked to receive? - Do you find it easy to come in to the clinic from the beginning? - Have you had any trouble adjusting to life with diabetes? - Have you been provided with any psychosocial support for your condition? Was this helpful? |
| **Maintenance** | - Barriers/challenges to adhering to appointments and prescribed medicine | - Do you come in regularly for all your appointments? Do you find it easy or difficult to do so? Why? (e.g. travel, time, stigma) - What is your experience when you come to the clinic? - Do you experience any difficulties when you are visiting the clinic for testing and treatment? - What could be done to make it easier for you to come to the clinic? - Do you take your medicines as often and regularly as you are prescribed to? Why? (e.g. don't think it's important) Do you find it easy or difficult to do so? Why? (e.g. difficult to remember) - Do you feel any pressure not to take your medicines (e.g. stigma from family or community?) - What could be done to make it easier for you to take your medications? - Do you find it easy to maintain a proper diet for your diabetes? - What challenges do you face? |
| **Efficacy** | - How coming to the clinic has affected patient's condition | - How do you think that coming to this clinic programme has affected your ability to live with your condition? - What would your condition be like if you didn't come to this clinic? Better or worse? Why? (i.e. are there any negative effects of this programme on individual's condition? Positive effects?) |
| **Thanks and close** | - Anything else to add - Questions - Thanks, feedback info | - Anything else to add on topic that we haven’t discussed today? - Any questions for me? |

**Appendix D: Topic guide for semi-structured interviews with diabetes health care providers.**

| **Key area** | **Themes** | **Question** |
| --- | --- | --- |
| **Introduction** | Study aim and agencies involved  Why invited to participate  Consent & any questions? |  |
| **Participant Background** | Getting to know each other + building rapport | Could you us a bit about yourself? *Prompt: e.g. professional, involvement in the diabetes service at Mwezo (and previously if relevant), what area live in, when you were first diagnosed with diabetes?* |
| **Reach** | Access  Barriers to testing and diagnosis Ways of reducing barriers | What are the key challenges to accessing testing for diabetes *e.g. knowledge, costs, time, availability of care, quality of care [expand], stigma etc.*  How could access to testing for diabetes be improved? Prompt: improve knowledge (e.g. *outreach, radio, health workers etc), improve availability of services, quality of services etc.* |
| **Adoption and implementation** | Information and support | What types of information are provided to patients when they are diagnosed with diabetes?  What sources of support are offered to patients when they are diagnosed with diabetes?  How acceptable do you think the diabetes treatment is for patients? *Prompt: e.g. quality, responsiveness, socially, culturally etc.* |
| **Maintenance** | Challenges  Supportive factors  To support adoption and implementation | What do you think are the main challenges facing diabetes patients here in terms of managing their diabetes? *Prompt: medicines/testing - time, costs, information, drug supply etc; dietary factor – knowledge, social/cultural pressures etc.*  What could be done to make it easier for diabetes patients to access care? *Prompt: e.g. information given – content and way it was delivered; costs; type and quality of care and support; [note: focus on role of the diabetes programme/services].* |
| **Efficacy** | Unintended consequences  Benefits | What are the benefits of the strengthened diabetes care programme in Mwezo? *Prompt: more efficient, less complications etc.*  What are negative consequences of the strengthened diabetes care programme in Mwezo? *Prompt: time, complexity, costs etc.*  What particular aspects of the revised programme have helped or hindered diabetes care? |
| **Thanks and**  **close** | Anything else to add  Questions  Thanks, feedback info | Anything else to add on topic that we haven’t discussed today?  Any questions for me?  Feedback again on how the discussion will be used and fed back. |
